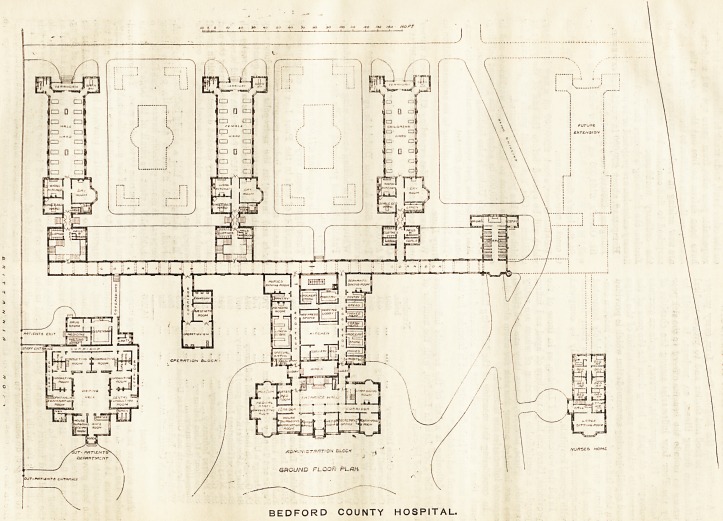# Hospital Construction

**Published:** 1898-09-10

**Authors:** 


### HOSPITAL CONSTRUCTION.

### BEDFORD COUNTY HOSPITAL.

We illustrate to-day the plans of the new general hospital erected for the county at Bedford, from the designs of Mr. H. Percy Adams, F.R.I.B.A. It will be seen that the blocks (the ends of which face south-east) are arranged for 16 beds each, and are respectively males, females, and children, the latter being of one storey, the two former of two stories each—surgical below, medical above. There is a separate ward for one bed attached to each pavilion, and there is a separate block, not shown on the plan, for isolation cases, bringing up the total number to 90 beds. The cost, including the nurses' home, is stated to be about £34,000, or about £370 per bed—a moderate sum, considering the ample accommodation provided. Aspect has been most carefully studied; the pavilions are placed 100 ft. apart to secure sun and air, and the day-rooms look south and west, with pleasant bays. The sanitary towers occupy the usual position at the ends of the wards, with balconies between them, and we note the provision of a separate w.c. for the nurses, opening out of the compartment reserved for the slop-sinks. There is a bath to each 17 beds, and a w.c. to eight or nine; but there are no lavatories, except, apparently, one in each bathroom. Each pavilion is completely furnished with its necessary adjuncts, has a separate staircase, and is separated by cross-ventilated lobbies from the main corridor, which separates it again from the administrative blocks. There is a separate block for operations, with a lavatory and dressing-room combined, and on either side lie the two blocks which provide for the out-patients and for administration. The former contains a large waiting-hall, with porch and ante-room, six consulting-rooms, four dressing-rooms, and a dispensary, all approached by well-lighted and convenient corridors, but the lavatories seem rather too closely connected to the building. The administration block is two-storeyed in front, and this portion contains the administrative rooms proper, while behind are the kitchens, larders, stores, &c., very completely arranged and served by two corridors, which connect them with the main corridor above referred to. It is not quite apparent why the w.c. and lavatory for the medical staff are contained in the kitchen block, when they might have been placed nearer the front building. The servants and nurses have dining-rooms placed respectively north and south of the kitchen block, while the staff dining-room is in the front, near the entrance hall. There is a chapel between the southern block and the site reserved for extensions, and the nurses' home lies in front of this, its arrangement being suitable and calling for no special comment. The wards have glazed brick walls up to a height of five feet, with hard cement above, and all the floors are of terrazzo. It is to be regretted that a view of the interior cannot be reproduced as well as the plans, for the appearance, with red brick walls and tiled roofs, would seem to be as picturesque as the plan is satisfactory.

BEDFORD COUNTY HOSPITAL.

## Figures and Tables

**Figure f1:**